# Impact assessment of Iran’s health technology assessment programme

**DOI:** 10.1186/s12961-018-0286-0

**Published:** 2018-02-22

**Authors:** Bahareh Yazdizadeh, Farideh Mohtasham, Ashraf Velayati

**Affiliations:** 1 Knowledge Utilization Research Centre (KURC), Tehran University of Medical Sciences, Tehran, Iran; 2 Shaheed Beheshti University of Medical Sciences, Tehran, Iran

**Keywords:** Health technology assessment, Health research impact assessment, Research payback

## Abstract

**Background:**

Following approximately 10 years from the beginning of Iran’s national Health Technology Assessment (HTA) programme, the present study aims to evaluate its success by examining the impact of HTA and identifying the determinant factors leading to the implementation of HTA report results.

**Methods:**

The triangulation method was employed herein. HTA reports were initially identified and their impact and determinant factors were then examined from the perspectives of both researchers (by preparing a questionnaire according to the Payback model and sending it to HTA principle investigators) and stakeholders (semi-structured interviews held with each HTA stakeholder). Simultaneously, the quality of the HTA reports was examined with relevant critical appraisal checklists.

**Results:**

The impact of 19 equipment technologies and four pharmaceutical technologies were assessed in this study. Twenty researchers replied (response rate, 86.96%) to the questionnaire on the impact of HTA reports from the researcher’s perspective. To assess the impact of HTA reports from the stakeholder’s perspective, seven policy-makers were chosen and interviewed as the main target audience.

The most common step taken to disseminate the results of the HTA projects was publication. Conducting the HTA had taught researchers and their colleagues’ new skills and had facilitated the securing of research grants from other organisations. Most reports had used the systematic review method but the relevant details had been scarcely presented regarding outcomes, costs and analysis. The greatest impact of HTA reports on decision-making had been on policy-makers providing and allocating finances. Barriers in stewardship, identification and prioritisation of topics, performance and dissemination of HTA results were the main barriers of implementing HTAs.

**Conclusions:**

In most aspects, the status of HTA impact reports need improvement. Thus far, the barriers and facilitators of the HTA programme in Iran have been investigated in other studies. These findings should be pooled to reach a solution that can be actively applied to the health system to improve the status quo of HTA in Iran.

**Electronic supplementary material:**

The online version of this article (10.1186/s12961-018-0286-0) contains supplementary material, which is available to authorized users.

## Background

The rapid dissemination of health technologies has exposed governments to challenges in the delivery of high-quality and effective care. Therefore, governments are increasingly obliged to manage limited resources through investing in services that have the best health outcomes. Health technology assessment (HTA) is the systematic assessment of the impact and characteristics of health system technologies. It is a multidisciplinary process that examines the medical, socioeconomic, developmental, distribution and utilisation issues of technology. HTA examines the direct and expected, indirect and unwanted, and short- and long-term results of technology, and reports clear unbiased data on the utilisation of health technology through a systematic method [1]. The goal of conducting HTA studies is to efficiently and effectively use healthcare services resources to safeguard the public health systems financial resources [2]. Therefore, by performing HTA, evidence-based data is provided to the health system’s policy- and decision-makers [3–5].

In Iran, HTA activities began in 2007 in the form of a secretariat following the authorities’ decision. From 2010, changes in the structure of the Ministry of Health and Medical Education (MOHME) led to the separation of the Deputies of Health and Curative Affairs. At the same time, the ‘HTA Office’ began its activities in the ‘Health Technology Assessment, Standardisation and Tariff Unit’ under the supervision of the Deputy of Curative Affairs with a new structure [6]. The vision of the HTA Office in Iran is to establish HTA in the health system, such that all the health system’s decision-making would be based on scientific evidence and on HTA reports [6].

The main goals of research in the health system are to identify the most effective way to organise, manage, finance and provide high-quality services, reduce medical errors, and improve patient safety [5–7]. According to significant studies on the investment in health, measuring the impact of such investments can help to increase accountability, identify ways to increase the impact and allocate appropriate research funding [7]. Therefore, health system research institutes are actively assessing the impact of their research [8]. Explaining the extent to which the results of prepared HTA reports meet the set goals and the assessment of the impact of reports may be helpful in identifying appropriate strategies to increase the effectiveness of HTA.

To date, various methods have been applied to investigate the impact of HTA reports and to evaluate the impact of health research programmes [8, 9]. These methods are (1) desk or evidence analysis, a common technique which involves a variety of activities, including analysis of documents, evidence and research project files, and in particular ISI cited documents (retracting information on the number of citations and the impact factor of the magazine); (2) questionnaires, usually employed as part of the impact assessment process allowing the views of all interested parties to be included; (3) interviews, performed either face to face or via telephone with all interested parties; and (4) detailed case study assessments, using a combination of the abovementioned methods, and therefore naturally more time consuming.

Following selection of the impact evaluation approach, a specific framework should also be employed. Various frameworks have been introduced to evaluate the effects of health research programmes, including the (1) Monetary Value framework, used to estimate the return on investment from research and evaluating direct and indirect costs of the study [8, 9]; (2) the Economic and Social Research Council framework used to examine the non-academic impact of socioeconomic research [10]; (3) the Dutch Academy of Science and Art framework used to assess the social impact of health studies [8]; (4) the Lavis et al. framework used for assessing the impact of health studies on politics (focusing on a specific target group) [11]; (5) Hailey’s organisational theory framework [12]; (6) the Knowledge Application Model [13]; (7) the impact model provided by the American agency of Healthcare and Research Quality [14]; and (8) the Payback framework [15]. This latter approach provides a comprehensive framework to assess the impact of health studies and classifies a wide range of potential health research benefits into different groups, as follows:

1. Knowledge production: It reflects the outputs resulting from the study (paper or any other printed documents).
2. The benefits include better targeting for future research, development of research skills, personnel and total research capacity, increased applicability of overseas study, and staff development/educational benefits.
3. Policy and administrative benefits include improving the databases required for policy and executive decisions, and other policy benefits resulting from research
4. Benefits to the health sector include cost savings in the provision of existing services, quality improvement in the service delivery process, enhancing the effectiveness of services (e.g. promoting health), justice (e.g. improving resource allocation at regional level), better targeting and access, revenue derived from intellectual property rights, and organisational development.
5. Broad economic benefits include the economic benefits derived from commercial exploitation of research and development innovations, and those gained from a healthy workforce and a reduction in the number of working days lost.

Given the widespread classifications within the Payback framework covering most of the requirements to measure impact, this framework was selected to evaluate the impact of HTA reports. Thus, the present study was conducted using the Payback analytical framework to evaluate the impact of HTA reports prepared during the period from September 2008 to September 2013, with a case study approach using a combination of questionnaire analysis and expert interviews.

## Methods

A mixed triangulation method was employed in the case study approach. HTA reports were initially identified, and then their impact and the determinant factors were then examined from the perspectives of the researchers and HTA stakeholders. Simultaneously, the quality of the HTA reports was examined with relevant critical appraisal tools.

All Iranian HTA reports that had been completed between September 2008 and September 2013 were included in this study. These reports had been published by MOHME’s HTA Office and were available in the form of six books.

### Identifying the impact of HTA reports from the researchers’ perspective

A questionnaire (Additional file 1) was designed by the research team, based on the Payback model, to examine the impact of HTA projects. In the Payback model, research impact includes the five domains of knowledge advancement, capacity-building, impact on decision-making, impact on health, and socioeconomic impacts [9]. However, since the assessment of health and socioeconomic impacts requires a considerable time to have elapsed since the completion of HTA, herein we investigated the first three impacts, i.e. knowledge advancement, capacity-building and impact on decision-making. The first part of the developed questionnaire consisted of two sections, namely general specifications (about the colleagues participating in the project, their affiliations, the date of project commencement and end, its budget and its adequacy, the preparation of part or all of the infrastructures required for the assessment through channels other than the project budget, the founder of the assessment) and stakeholder engagement (the stakeholders’ engagement in the project, their engagement types and their level of participation). In addition, a number of questions on the probable barriers of HTA utilisation were also included in the questionnaire.

Knowledge advancement was investigated by the number of articles produced on the basis of HTA (in the questionnaire, the researchers were asked about the articles published; the research team then examined the status of those articles’ indexing in PubMed and Scopus and their number of citations). Capacity-building impact was also investigated by garnering points for making an impact, acquisition of new skills, securing budget for future research, utilisation of assessment results for defining future research studies, whether the HTA was a thesis or not, and utilisation of financial resources to strengthen the resources of the research organisation in which the researcher works in. Finally, the impact of HTA reports on decision-making in the questionnaire was investigated by defining the stakeholders that could be affected by HTA. These stakeholders included technology providers (including physicians, pharmacists, nurses, the country’s Medical Council, Food and Drug Administration, etc.); public companies importing or producing technology; private companies importing or producing technology; policy-makers providing and allocating finances to technology, including the Offices of Insurance and Medical Equipment; the Supervisory and Financing Unit of Curative Affairs; the Iranian Parliament’s Commission of Health and Curative Affairs; MOHME’s Policy-making Council; policy-makers of MOHME’s Clinical Governance and Hospital Management Unit; policy-makers at MOHME’s Standardisation and Tariff Office; policy-makers regulating technology-related research needs, including Deputies of Research and Technology of Universities of Medical Sciences; policy-makers regulating technology-related educational needs, including Deputies of Education; and policy-makers regulating innovation-related research needs, including Deputies of Research and Technology of Universities of Medical Sciences.

The researchers were then asked about the use of HTA results in documents relevant to policy-making, such as systematic reviews, clinical guidelines or public health guidelines, patient decision aids, policy briefs, policy documents, guidelines, and/or legislations of executive organisations. Furthermore, they were questioned on the utilisation of assessment results in book compilation, development of educational content for professional groups (continuing education and/or academic students’ education), policy-making outside the health system and local policy-making.

At the end of the questionnaire, an open question was asked to the researcher: ‘If your HTA has not been utilised as expected, what do you think the reasons are for its inappropriate utilisation?’

After calling the main researcher of the project through telephone and explaining the objective of the study, the questionnaire was sent through e-mail and reminders were also sent. To ensure quality assurance and to examine the feasibility of the questionnaire, it was sent to two principle investigators (PIs) in the form of a pilot study and their opinions were implemented in the final version of the questionnaire.

### Identifying the impact of HTA reports from the target audience’s perspective

At this stage, we wanted to identify impacts of HTA reports and barriers of implementation of HTAs from the target audience’s perspective. To this end, the main stakeholders of Iran’s HTA reports were identified from Yazdizadeh et al.’s study [16], which identified policy-makers at MOHME’s Standardisation and Tariff Office, the Medical Equipment Office, the Food and Drug Administration and insurance organisations as the main target audiences of HTAs in Iran. Thus, semi-structured interviews were held with their representatives upon purposive sampling of interviewees.

The interviews were held with individuals who consented to participate in the study in a place that was calm and quiet (in the opinion of the interviewee). A note-taker was also present in the interview along with the interviewer. The interview was from 45 minutes to 1-hour long. The general questions were ‘how do you interact with the HTA Office?’ and ‘how are you informed of the HTA reports results?’. Thereafter, the list of reports that were included in this study (HTAs conducted between September 2008 and September 2013) was given to the interviewee to see whether or not they had been informed of the report results involving their organisations and what was the impact of the report.

The interviews were recorded with a voice recorder, and the audio file of each participant was saved with a special code and eventually transcribed. For data analysis, the interviews were first transcribed and the transcriptions were then studied. The primary categories (open coding) were identified and the codes were grouped and axial codes identified (themes or axial coding). Eventually, the main category was identified (selective coding). The interviews were studied by two individuals independently; coding was also performed independently. Then, the codes, themes and categories were reviewed and consensus was reached during consecutive sessions. Data underwent thematic analysis, such that two categories, namely the status of utilisation of HTA results and the barriers toward utilisation of HTA results (at the stages of identification and prioritisation of topics, performing and disseminating HTA), were determined through a deductive approach. The themes and codes were extracted from the interviews through an inductive approach.

### Examining the quality of HTAs

The quality of HTAs was investigated as an aspect of knowledge advancement. HTA reports consist of two main sections, namely systematic reviews and economic evaluation studies. Therefore, in addition to the checklists that examine HTA reports on an overall basis, we also used systematic reviews and economic evaluation checklists (Table 1).

**Table 1:** The checklists used to assess the quality of reports

The checklists used to assess the quality	Type of study
Preferred Reporting Items for Systematic review and Meta-Analysis (PRISMA) 2009 Checklist [21]	Systematic review
International Network of Agencies for Health Technology Assessment (INAHTA) [18] The HTA Core Model® [17]	Health technology assessment
The Quality of Health Economic Studies Instrument (QHES) [19] Drummond checklist (Drummond 1996) [20]	Economic evaluation

To increase the validity and reliability of the reports’ quality assessment, two HTA reports were selected and the initial checklists were evaluated by two colleagues separately. Then, during a session with a third colleague, the responses to the checklists’ questions were examined and uncertainties were clarified. Furthermore, quality control was performed by assessing the quality of all the reports by two individuals; cases of disagreement were examined by a third person and the final decision was made.

## Results

Overall, 23 HTAs were included in this study, 19 of which involved equipment technologies and four involved pharmaceutical technologies.

### The impacts of HTA reports from the researchers’ perspective

#### General specifications of HTA reports: identifying the impact of HTA reports from the researchers’ perspective questionnaire

Of the questionnaires sent to the PIs of the 23 conducted HTAs, 20 researchers replied (response rate, 86.96%). Of these 20 reports, three involved pharmaceutical-related technologies and 17 involved medical equipment-related technologies. Eighteen projects had reported the beginning and end of their project’s date; the mean duration of the reports was 1 year.

All 20 HTA reports had been financially supported by the HTA Office. The project’s budget varied from US$1170 to US$2580, and the average budget was US$1700. The researchers of 12 reports (60%) declared that the financial resources were adequate; however, the researchers of 8 reports (40%) were dissatisfied with the amount of resources allocated.

In the PI’s opinion, political factors had no effect on the delivery of 19 reports (one researcher had not answered this question).

Of the 17 equipment-related reports, five cases (29.4%) had selected all the recommended options as their stakeholders. Three cases (17.6%) had not selected any of the options as their stakeholders. Of the three pharmaceutical reports, two cases (66.67%) had chosen all the recommended options as their stakeholders (Table 2).

**Table 2:** Distribution of stakeholders for each type of technology according to the researchers’ perspective

Stakeholders	Equipment technologies (17 cases)	Pharmaceutical technologies (3 cases)	Total technologies (20 cases)
Technology providers^a^	13 (76.5%)	3 (100.0%)	16 (80.0%)
Companies importing or producing technology	11 (64.7%)	3 (100.0%)	14 (70.0%)
Policy-makers providing and allocating finances to technology^b^	13 (76.5%)	3 (100.0%)	16 (80.0%)
Policy-makers regulating technology-related research and innovation needs^c^	11 (64.7%)	2 (66.7%)	13 (65.0%)
Policy-makers regulating technology-related educational needs including Deputies of Education	5 (29.4%)	2 (66.7%)	7 (35.0%)
Public, patient associations, etc.	8 (47.1%)	3 (100.0%)	11 (55.0%)

^a^Include physicians, pharmacists, nurses, the Medical Council, Food and Drug Administration, etc.

^b^Includes the Offices of Insurance and Medical Equipment, the Supervisory and Financing Unit of Curative Affairs, the Iranian Parliament’s Health Commission, MOHME’s Policy-making Council, policy-makers at MOHME’s Clinical Governance and Hospital Management Unit, policy-makers at MOHME’s Standardisation and Tariff Office

^c^Includes Deputies of Research and Technology, Universities of Medical Sciences, etc.

#### Stakeholder engagement: identifying the impact of HTA reports from the researchers’ perspective questionnaire

In response to the question on stakeholder engagement rate in the development of HTA reports, it was indicated that technology providers and policy-makers providing and allocating finances for technology had the highest rate of participation in preparing the 20 HTA reports. In response to the type of stakeholder engagement, it was shown that the highest rate of stakeholder engagement was during the extraction of conclusions (Table 3).

**Table 3:** The rate and method of active participation of stakeholders in the development of health technology assessment reports

Type of stakeholders	Are they stakeholders?^a^	Have they participated?^b^	At what level of participation?^c^			
			Proposal preparation	Execution	Analysis	Conclusion
Technology providers	16 (80.0%)	15 (80.0%)	8 (53.3%)	9 (60.0%)	7 (46.7%)	9 (60.0%)
Companies importing or producing technology	14 (70.0%)	5 (35.7%)	1 (20.0%)	0	2 (40.0%)	3 (60.0%)
Policy-makers providing and allocating finances to technology	16 (80.0%)	11 (68.8%)	4 (36.4%)	5 (45.5%)	1 (9.1%)	7 (63.6%)
Policy-makers regulating technology-related research and innovation needs	13 (65.0%)	3 (66.6%)	2 (66.6%)	0	0	1 (33.3%)
Policy-makers regulating technology-related educational needs	7 (35.0%)	0	0	0	0	0
Public, patient associations, etc.	11 (55.0%)	0	0	0	0	0

^a^The numerator is the number of reports with a ‘yes’ response; the denominator is the total number of reports

^b^The numerator is the number of reports with a ‘yes’ response; the denominator is the total number of stakeholders

^c^The numerator is the number of reports with a ‘yes’ response; the denominator is the total number of participations

#### Knowledge advancement: identifying the impact of HTA reports from the researchers’ perspective questionnaire

The most common strategies taken to disseminate the results of the HTA projects were the publication of articles in domestic scientific journals, delivery of the complete or summarised reports to their potential stakeholders, publication of articles in international scientific journals, and presentation in domestic conferences, workshops and seminars (Table 4). It must be noted that, although all 20 reports had been published in the form of a book by MOHME and their full texts were available in the HTA Office website, less than half of the researchers were aware of this and declared it as one of the steps taken to disseminate their results.

**Table 4:** Strategies taken to implement health technology assessment (HTA) results

Strategies taken	Number of reports taking this step
The publication of articles in domestic scientific research journals	12 (60.0%)
Publication of articles in international scientific research journals	7 (35.0%)
Presentation in domestic conferences, workshops and seminars	7 (35.0%)
Presentation in international conferences, workshops and seminars	3 (15.0%)
Delivery of the complete or summarised reports to their potential stakeholders	9 (45.0%)
Displaying the complete or summarised reports in the website to allow access to potential stakeholders	5 (25.0%)
Dissemination of HTA results in non-scientific publications (such as magazines or newspapers read by the public)	1 (5.0%)
Dissemination of results in newsletters and bulletins (such as intra-organisational publications that publish the scientific and non-scientific news related to that organisation)	0
Presenting the assessment results to media journalists (radio, television) and/or giving interviews	0
Setting up meetings with potential stakeholders to introduce the assessment results	4 (20.0%)
Development and delivery of results in a language appropriate to the target audiences (such as simple writings for patients and/or the public, short reports for managers and authorities)	2 (10.0%)
Other steps that lead to the delivery of assessment results to their target audiences	1 (5.0%)

Of the 20 HTA reports, 15 articles (three articles in Persian and 12 in English) have been published (these articles are from 12 HTA reports; another eight reports have not published any articles). Three HTA reports had published two articles in different journals under different topics. Among the 12 English articles, six had been indexed in PubMed and Scopus, two had been indexed in Scopus alone, and three had been indexed in PubMed alone. The year of publication varied from 2011 to 2016. The mean citation rate of the 12 articles was 2.42. One article had eight citations, one had four, two articles had three, five had two, one had one, and the remainder had no citations (the indices and citations were checked in July 2016).

#### Capacity-building: identifying the impact of HTA reports from the researchers’ perspective questionnaire

All 20 PIs stated that conducting the HTA had taught them and their colleagues’ new skills. Other factors that had contributed to capacity-building were facilitating the securing of research budgets from other organisations (inside or outside the health system) and defining future research projects (Table 5).

**Table 5:** The capacity-building impact of health technology assessment reports from the researchers’ perspective

The dimensions of capacity-building	The number of reports that included this dimension		
	Yes	No	No response
The project had an impact on decision-making and had garnered points from MOHME	1 (5.0%)	19 (95.0%)	0
The co-investigators have acquired new skills (such as systematic review and/or economic evaluation) in order to design future studies	20 (100.0%)	0	0
The project facilitated the securing of research grants from other organisations (inside or outside the health system)	9 (45.0%)	10 (50.0%)	1 (5.0%)
The results of this project will be utilised for defining future research projects	9 (45.0%)	5 (25.0%)	6 (30.0%)
The project or part of the project was an academic thesis	2 (10.0%)	15 (75.0%)	3 (15.0%)
Part of the project costs had been used to strengthen the organisation’s research resources	1 (5.0%)	15 (75.0%)	4 (20.0%)
Part or all of the infrastructures required for this project had been provided by mechanisms other than the projects own costs	2 (10.0%)	15 (75.0%)	3 (15.0%)

#### The impact of HTA reports on decision-making: identifying the impact of HTA reports from the researchers’ perspective questionnaire

Regarding the impact of HTA reports on decision-making, the researchers believed that the greatest impact had been on policy-makers providing and allocating finances. Companies importing or producing technology and policy-makers regulating technology-related research and innovation needs had been least affected by the HTA reports (Table 6); 84.2% of PIs expected their HTA results to be utilised in future decision-making.

**Table 6:** The impact of health technology assessment reports on decision-making from the researchers’ perspective

Stakeholders	Change created				The rate of impact of the report and decision-making				
	Yes	No	I do not know	No response	< 25%	25–50%	50–70%	> 70%	No response
Technology providers	3 (15.0%)	3 (15.0%)	12 (60.0%)	2 (10.0%)	1 (5.0%)	1 (5.0%)	1 (5.0%)	0	17 (85.0%)
Companies importing or producing technology	2 (10.0%)	7 (35.0%)	10 (50.0%)	1 (5.0%)	0	0	0	1 (5.0%)	19 (95.0%)
Policy-makers providing and allocating finances	7 (35.0%)	4 (20.0%)	7 (35.0%)	2 (10.0%)	0	2 (10.0%)	3 (15.0%)	0	15 (75.0%)
Policy-makers regulating technology-related research and innovation needs	3 (15.0%)	6 (30.0%)	9 (45.0%)	2 (10.0%)	0	1 (5.0%)	1 (5.0%)	0	18 (90.0%)
Policy-makers regulating technology-related educational needs	0	9 (45.0%)	8 (40.0%)	3 (15.0%)	0	0	0	0	20 (100.0%)
Public, patient associations, etc.	0	3 (15.0%)	2 (10.0%)	15 (75.0%)	0	0	0	0	20 (100.0%)

Regarding the identification of documents used in decision-making, the PIs stated that the results of the HTAs had been used in seven systematic reviews and five policy briefs. However, after reviewing the results, we found that they had considered the systematic reviews published by themselves as the applicable results of their own studies, whereas, in fact, only two of the project results had been used in review studies. With respect to policy briefs, we found that the researchers actually meant ‘executive summaries’ that were delivered along with the final reports. Therefore, no policy brief had been prepared from any of the projects assessed.

As mentioned earlier, all 20 reports have been published in the form of a book by the HTA Office. However, only five researchers were aware of this and reported it as one of the steps taken to implement their results (Table 7).

**Table 7:** Utilisation of health technology assessment reports in documents related to decision-making

Type of document	Yes	No	I do not know	No response
Systematic reviews	2 (10.0%)	5 (25.0%)	4 (20.0%)	4 (20.0%)
Service delivery guidelines (clinical guidelines or public health guidelines)	1 (5.0%)	5 (25.0%)	7 (35.0%)	7 (35.0%)
Educational content for patient and/or the public (patient decision aids, etc.)	1 (5.0%)	6 (30.0%)	5 (25.0%)	8 (40.0%)
Policy brief (policy brief is a documentation that is prepared to help make decisions about the possible options of policy-making and includes scientific evidence on the advantages and disadvantages of various policy-making options)	0	4 (20.0%)	4 (20.0%)	7 (35.0%)
Policy documents, guidelines and/or executive organisations’ legislations	0	4 (20.0%)	9 (45.0%)	7 (35.0%)
Book compilation	5 (25.0%)	14 (70.0%)		1 (5.0%)
Development of educational content for professional groups (continuing education and/or education of academic students)	0	19 (95.0%)		1 (5.0%)

None of the 20 HTA results had been utilised in policy-making outside the health system (directly or indirectly), whereas 13 researchers (65%) gave a negative response and 4 (20%) said it was not applicable. One PI (5.0%) stated that their HTA results had been utilised in their local context policy-making, whereas 11 individuals (55%) gave negative responses and 7 (35%) said it was not applicable.

#### Barriers of implementation of HTAs: identifying the impact of HTA reports from the researchers’ perspective questionnaire

At the end of the questionnaire, the factors affecting the implementation of reports as mentioned by the researchers included the scarcity of evidence, the low budget allocated to research execution, the imperfect procedure of legislation, execution and review of HTA projects, inappropriate professional ties between those prioritising HTA topics with executive or end-user units, inappropriate notification of the HTA Office and lack of its familiarity and interaction with stakeholders, the difficulty associated with the publication of HTA articles in well-known journals (this very issue lowers the stakeholders’ trust and the quality of the reports), lack of executive assurance for utilisation of HTA projects’ results as an important part of decision-making at the macro level, lack of dissemination of results in the user’s language, and conflicts of interests between the policy-maker, importer and user of technology.

### The impact of HTA reports from the target audience’s perspective

Semi-structured interviews were held with the stakeholder representatives identified for these technologies; seven individuals were selected from the Article 20 Commission (MOHME’s Deputy of Curative Affairs has the Article 20 Commission in the field of Medical Equipment; in fact, they have the license to import expensive Medical Equipment), the HTA Office’s Insurance unit, MOHME’s Standardisation and Tariff Office, the Medical Equipment Office, Iranians Health Insurance Organisation, and the Higher Council of Insurance and Medical Services. The impact of HTA reports on stakeholder organisations varied.

The Article 20 Commission is where the HTA reports can be said to have definitely had an impact. As mentioned earlier, all medical equipment must receive a license from the Article 20 Commission before they can be imported into the country for the first time. We observed that, for six cases of medical equipment technology, the HTA reports results convinced the Article 20 Commission to only allow the entry of a limited number of each technology. Moreover, when the equipment-importing companies had been informed of the HTA results, they had decided to cancel the imports themselves. It also appears that induced demand of novel technologies has largely been controlled due to the HTA reports and the impact they have had on the Article 20 Commission’s decisions. However, regarding other stakeholder organisations, the impact of HTA reports on decisions has not been very clear, and what needs to be improved is the active dissemination of report results to stakeholders.

Some organisations claimed to have used the results when they had had access to them. Some of the stakeholders’ statements are as follows:

“*We act accordingly when we receive a report, unless the HTA development process takes very long and they are under a lot of pressure, in which case they decide until the result arrives.*”

“*If the HTA result is negative, i.e. it does not qualify for being in the country’s health domain, then no-one will talk about it anymore.*”

“*I am aware of at least two cases in which they have acted exactly according to the HTA report.*”

“*If we could be certain that MOHME would give us information we would wait, but we have had no HTA so far. We are legally bound to send an HTA request to MOHME. However, nothing has happened so far; so usually, when we know something is a medical and societal need we don’t wait for MOHME’s response.*”

Nonetheless, one of the interviewees from an insurance organisation said,

“*I haven’t seen MOHME representatives consult HTA* [in the Higher Insurance Council that is formed in the Ministry of Social Welfare, which is responsible for allowing the entry of technology into the insurance services package].”

There were also implementation barriers from the target audience’s perspective. The ‘barriers’ category will be described in four themes; barriers in stewardship, identification and prioritisation of topics, performance and dissemination of HTA results.

#### HTA programme stewardship

One of the most important issues mentioned in the interviews was that the service-providing organisation was separate from the fund-providing organisation. In Iran, MOHME is responsible for delivering health services, but the costs of service delivery are assigned to another ministry, called the ‘Ministry of Social Security and Welfare’, by which insurance organisations are supervised. According to one participant,

“*Decision-making regarding the benefit package and its stewardship are defragmented in the country.*”

Another major issue addressed by the participants was the structural barrier. According to one participant,

“*It was mentioned* [in the Sixth Development Plan] *that insurance organisations should be integrated* [into MOHME]*, but they haven’t been yet. If one organisation goes forward the same problems arise again.*”

Furthermore, one interviewee believed that, as long as the HTA Office is under MOHME’s supervision and is financially supported by it, it cannot act independently. In their opinion,

“*All the stakeholders should be involved in the provision of resources for the project,*” and “*if HTA becomes independent then everyone will begin to trust it.*”

#### Identification and prioritisation of topics

One of the most important barriers observed in this study was the lack of engagement of HTA stakeholders in determining the topics and prioritising them for assessment; this issue was observed more often in some stakeholders.

“*Not all stakeholders and end-users are involved in HTA topic selection, such as insurance organisations and service delivery centres.*”

The lack of engagement of stakeholders at this stage of assessment leads to other problems, one of which is the lack of consistency between stakeholder organisations’ priorities and the HTA Office’s priorities. This problem is seen more prominently among stakeholders outside MOHME. According to one of the participants,

“*The Higher Insurance Council’s agenda does not match that of MOHME’s.*”

Subsequently, the assessments made in MOHME are not mentioned in the Higher Insurance Council (HIC) at all, because they are not on the agenda in the first place. Furthermore, insurance organisations are faced with a large volume of services that require assessment. However, the HTA Office only performs assessments for novel technologies that enter the market. According to one of the participants,

“*At least the needs assessment and prioritisation of topics should be common.*”

Another important point that was mentioned by one interviewee was that some stakeholders are unaware of the priority-setting criteria and procedures in MOHME. By damaging the transparency principle, the HTA programme’s success could face serious challenges.

#### Performing the HTA

One of the barriers observed in this study was the lack of stakeholders’ involvement in writing the HTA proposals. Subsequently, this lack of involvement resulted in, for example, PICO (population, intervention, compare and outcome) not being set according to the real circumstances and the users’ needs in the systematic review of the cost-effectiveness section. Therefore, such results cannot be utilised in the real world. According to one of the interviewees,

“*The expectations we have from cost effectiveness is the least expectation we have from HTA. For example, I as an insurance organisation would like to cover certain services; so I’ll do its economic evaluation separately. You might say that it is cost effective, but adapting it to insurance resources, equity, and the other items in insurance is a whole new story.*”

Another barrier mentioned in this section was the lack of belief in the quality of the HTAs by MOHME’s managers and stakeholders outside MOHME. Such a phenomenon does not allow managers to defend the assessment results with certainty.

Another problem mentioned was that the assessment process is too lengthy, which is longer than the duration required for decision-making. According to one interviewee,

“*I think the problem is not the procedure of the project, i.e. the duration is logical, and perhaps a shorter duration would not be logical. But it is the bureaucracy, the call for proposals, and referral of the project to individuals and announcement of the results that takes long.*”

Shortage of human resources for conducting the HTA was another barrier that was mentioned by the participants of this study.

#### Dissemination of HTA

According to the interviewees, the dissemination of results is not desirable. HTA reports and their summaries are put on display in MOHME’s HTA Office’s website, however, this is a type of inactive dissemination; the active dissemination of results demands greater attention.

Even when the clients have ordered the assessment themselves they are never informed of the results. Some of the statements in this regard are as follows:

“*No HTA …. Has been delivered to me.*”

*“We’ve requested at least 5 to 6 cases of HTA that have not been delivered to us.*”

“*We’ve never seen an HTA report to be able to utilise it.*”

“*The person who had done the HTA brought us the results.*”

The method of delivering the report results in stakeholders’ meetings is not desirable. For example, the assessment result is directly mentioned in the HIC, which is not the right time and place for its expert review; the inappropriate opposition of a single member (which stems from lack of trust in HTA results in comparison to international studies) can change the direction of the meeting. One interviewee believed that, if the results of the assessment were first examined in a session with the experts and then put forth in the final HIC meeting, they would more likely be utilised.

The interesting point was MOHME’s and the insurance organisations’ negative perspectives. According to one interviewee “*lack of dissemination of results is because of conflicts of interests at the level of MOHME, companies, etc*.” This indicates the deep and negative beliefs of extra-organisational stakeholders towards MOHME. Other relevant statements are as follows:

“*They have reports* [MOHME]*, but they don’t give them to us.*”

“*I think MOHME is trying to show that insurance organisations are inefficient.*”

“*The decisions made by the HIC Secretariat members are faced with fewer conflicts. Even if there are any they are negligible; they decide more freely as compared to those from the other side* [MOHME].”

“*I think in practice nothing special happens in MOHME. Even if something special does happen it’s for people who do something and have been granted a budget and will benefit from it; nothing happens for the nation.*”

“*Drug affairs have been handed over to the Food and Drug Organisation; they function much more actively; equipment-related affairs are still dependent on MOHME.*”

“*About HTA, I know that, for example, we had asked for a drug; it went under HTA and since it was not cost-effective the authority in MOHME who was supposed to approve it told the researcher to study its cost effectiveness, and the researcher refused to do so.*”

Furthermore, MOHME does not have a positive outlook toward insurance organisations. As stated by the interviewees,

“*Our Insurance System is faulty; for example, if the policy-maker prefers a service that does not have a lucrative HTA, it may be given preference over a service that has had a better HTA.*”

“*They say that when MOHME announces something we are OK with it, but we know that this is not the case.*”

### The quality of HTA projects

In this study, critical appraisal was done with five checklists. The quality assessment results of the reports are described in detail for each different checklist below.

#### The quality of HTA projects based on the Core Model Checklist

In the beginning we used all the questions of the Core Model [17]: Medical and Surgical Interventions (2.0) checklist for the assessment. However, since responding to some of the questions was not possible, the list of questions was finalised according to Table 8. As shown in Table 8, it is clear that all the reports abided by the principle of certainty and clarity in presenting their results and determined which diseases that technology affects. However, none of the reports presented a clear introduction of the research team (specialty, degree, affiliation) and have only mentioned their names. Some of the questions had not been responded to in any of the reports, e.g. determining the harm and hazard of using the technology for specialists, public and the environment, determining the direct effect of the technology on quality of life, the patients daily activities and returning to work, determining the effect of utilising the technology on human dignity and its relationship with the patients’ religious and cultural beliefs, determining the hazardous effects of the technology through different brands, and the methods of quality assurance of utilisation of the technology. Most of the reports were focused on determining the target population of the technology, accurate and scientific definition of the disease targeted by the technology, the risk factors, causes and side effects, disease symptoms and stages, the necessity of using the technology in comparison to similar existing technologies, and the cost effect of using the technology.

**Table 8:** Results of report assessments based on the HTA Core Model for Medical and Surgical Interventions

No.	Selective questions from the Core model	The number of reports that had taken this step
1	Which disease/health problem/potential health problem will the technology be used for?	23 (100.0%)
2	What, if any, is the precise definition/characterisation of the target disease? Which diagnosis is given to the condition and according to which classification system (e.g. ICD-10)?	15 (65.2%)
3	What is the natural course of the condition?	17 (74.0%)
4	What are the consequences of the condition?	21 (91.3%)
5	How many people belong at the moment (will belong) to the specific target group (describe according to sex, age)?	11 (47.8%)
6	What is the burden of disease (mortality, disability, life years lost)?	4 (17.4%)
7	How much is the technology being used?	12 (52.2%)
8	Describe the variations in use across countries/regions/settings, if any?	8 (34.8%)
9	How is the disease/health condition currently being managed?	4 (17.4%)
10	According to published algorithms/guidelines (if any), how should the condition be managed?	3 (13.0%)
11	What are the other evidence-based alternatives to the current technology, if any?	18 (78.3%)
12	Which approval status has the technology received in other countries, or from international authorities?	6 (26.1%)
13	Why is this technology used?	17 (73.9%)
14	Who will apply this technology?	11 (47.8%)
15	What is the place and context for utilising the technology?	8 (34.8%)
16	What material investments are needed to use the technology?	6 (26.1%)
17	What kind of qualification, training and quality assurance are needed for the use or maintenance of the technology?	3 (13.0%)
18	What kind of training and information are needed for the patients receiving or using this technology and their families?	2 (8.7%)
19	What kind of harms can use of the technology cause to the patient and what are the incidence, severity and duration of harms?	7 (30.4%)
20	What is the dose that can harm the patients?	3 (13.0%)
21	What is the timing of onset of harms to patients: immediate, early or late?	2 (8.7%)
22	What kind of occupational harms can occur when using the technology?	0
23	What kind of environmental risks may use of the technology cause?	0
24	How does the safety profile of the technology vary between different generations, approved versions or products?	0
25	How can one reduce safety risks for patients (including technology-, user- and patient-dependent aspects)?	1 (4.4%)
26	What is the mortality related to the technology studied?	1 (4.4%)
27	How does the intervention modify the severity and frequency of symptoms and findings?	3 (13.0%)
28	How does the technology modify the need for hospitalisation?	3 (13.0%)
29	How does the technology modify the need for other technologies and use of resources?	2 (8.7%)
30	What is the effect of the intervention on return to work?	0
31	How does the use of the technology affect activities of daily living?	0
32	What is the effect of the intervention on health-related quality of life?	0
33	Can the technology challenge religious, cultural or moral convictions or beliefs of some groups or change current social arrangements?	0
34	What are the consequences of implementing/not implementing the technology on justice in the healthcare system? Are principles of fairness, justness and solidarity respected?	2 (8.7%)
35	Does the implementation or use of the technology affect human dignity?	0
36	Would the patient be willing to use the technology again?	4 (17.4%)
37	What are the unit costs of the resources used when delivering the assessed technology?	15 (65.2%)
38	What are the unit costs of the resources used when delivering the assessed comparators?	12 (52.2%)
39	What is the impact of the technology on indirect costs?	14 (60.9%)
40	What is the incremental cost-effectiveness ratio?	11 (47.8%)
41	In what way is the quality assurance and monitoring system of the new technology organised?	0
42	What kind of staff, training and other human resources is required?	1 (4.4%)

#### The quality of HTA projects based on the International Network of Agencies for Health Technology Assessment (INAHTA) Checklist

The first five questions of the INAHTA checklist [18] regard the initial data (author, content, conflict of interests, etc.), and were not presented appropriately by most of the assessment reports. Why the assessment was conducted (questions 6, 7, 8 and 9), questions about policy-makers, research questions, and description of the technology had all been clearly stated in most of the reports, however, a clear picture of the domain assessed was not presented. With regards to how the assessment was conducted (questions 10–24), most reports had presented proper details of the processes that had taken place. Questions 24–28, which examine the domains of technology assessment in reports, also showed that most reports had assessed technologies cost-wise and few had dealt with other domains of assessment. Regarding the presentation and interpretation of results (questions 29–33), we observed that most reports had presented their results with clarity; however, few had ideas for future research (Table 9).

**Table 9:** Results of report assessments based on the International Network of Agencies for Health Technology Assessment (INAHTA) checklist

No.	INAHTA’s questions	The number of reports that had taken this step
1	Appropriate contact details for further information?	0
2	Authors identified?	2 (8.7%)
3	Statement regarding conflict of interest?	1 (4.4%)
4	Statement on whether report externally reviewed?	0
5	Short summary in non-technical language?	13 (56.5%)
6	Reference to the policy question that is addressed?	16 (69.6%)
7	Reference to the research question(s) that is/are addressed?	19 (82.6%)
8	Scope of the assessment specified?	6 (26.1%)
9	Description of the assessed health technology?	20 (86.9%)
10	Details on sources of information and literature search strategies provided? Search strategy	19 (82.6%)
11	Details on sources of information and literature search strategies provided? Databases	23 (100.0%)
12	Details on sources of information and literature search strategies provided? Year range	18 (78.3%)
13	Details on sources of information and literature search strategies provided? Language restriction	16 (69.6%)
14	Details on sources of information and literature search strategies provided? Primary data	17 (73.9%)
15	Details on sources of information and literature search strategies provided? Other kinds of information resources	10 (43.5%)
16	Details on sources of information and literature search strategies provided? Complete reference list of included studies	16 (69.6%)
17	Details on sources of information and literature search strategies provided? List of excluded studies	6 (26.1%)
18	Details on sources of information and literature search strategies provided? Inclusion criteria	17 (73.9%)
19	Details on sources of information and literature search strategies provided? Exclusion criteria	15 (65.2%)
20	Method of data extraction described?	12 (52.2%)
21	Critical appraisal method (for quality assessment of the literature) described?	13 (56.5%)
22	Method of data synthesis described?	5 (21.7%)
23	Results of the assessment clearly presented, e.g. in the form of evidence tables?	19 (82.6%)
24	Medico-legal implications considered?	2 (8.7%)
25	Economic analysis provided?	14 (60.9%)
26	Ethical implications considered?	2 (8.7%)
27	Social implications considered?	2 (8.7%)
28	Other perspectives (stakeholders, patients, consumers) considered?	3 (13.0%)
29	Clear presentation of the results (absolute and relative values?)	17 (73.9%)
30	A clear interpretation of the results?	19 (82.6%)
31	Findings of the assessment discussed?	14 (60.9%)
32	Conclusions from assessment clearly stated?	20 (86.9%)
33	Suggestions for further action?	5 (21.7%)

#### The quality of HTA projects based on the Quality of Health Economic Studies (QHES) Checklist

This is a 16-question checklist that assesses the quality of economic studies with a ‘yes’ and ‘no’ reply format and calculates the total score from 100 [19]. Since this checklist had been chosen to assess the economic evaluation section of the reports, only those reports that had this section were assessed by it (of 23 reports, nine were systematic reviews and only one had used costing in its economic section and lacked a specific method for its economic evaluation). After calculating the scores of 13 reports with the QHES checklist, we observed that the reports’ scores ranged from 38 to 87 with a mean of 72 (SD 15.25). A few of the reports had determined the perspective of analysis (30.77%) and examined the possible errors (38.46%) (Table 10).

**Table 10:** Assessment results based on the Quality of Health Economic Studies Instrument (QHES) Checklist

No.	QHES questions	Weightings	The number of reports that had taken this step
1	Was the study objective presented in a clear, specific and measurable manner?	7	12 (92.3%)
2	Were the perspective of the analysis (societal, third-party payer, etc.) and reasons for its selection stated?	4	4 (30.8%)
3	Were variable estimates used in the analysis from the best available source (i.e. randomised control trial – best; expert opinion – worst)?	8	11 (84.6%)
4	If estimates came from a subgroup analysis, were the groups pre-specified at the beginning of the study?	1	4 (30.8%)
5	Was uncertainty handled by (1) statistical analysis to address random events, (2) sensitivity analysis to cover a range of assumptions?	9	10 (76.92%)
6	Was incremental analysis performed between alternatives for resources and costs?	6	11 (84.6%)
7	Was the methodology for data abstraction (including the value of health states and other benefits) stated?	5	7 (53.8%)
8	Did the analytic horizon allow time for all relevant and important outcomes? Were benefits and costs that went beyond 1 year discounted (3–5%) and justification given for the discount rate?	7	4 (30.8%)
9	Was the measurement of costs appropriate and the methodology for the estimation of quantities and unit costs clearly described?	8	11 (84.6%)
10	Was the primary outcome measure(s) for the economic evaluation clearly stated and was the major short-term justification given for the measures/scales used?	6	8 (61.5%)
11	Were the health outcomes measures/scales valid and reliable? If previously tested valid and reliable measures were not available, was justification given for the measures/scales used?	7	7 (53.8%)
12	Were the economic model (including structure), study methods and analysis, and the components of the numerator and denominator displayed in a clear, transparent manner?	8	13 (100.0%)
13	Were the choice of economic model, main assumptions, and limitations of the study stated and justified?	7	9 (69.2%)
14	Did the author(s) explicitly discuss direction and magnitude of potential biases?	6	5 (38.5%)
15	Were the conclusions/recommendations of the study justified and based on the study results?	8	13 (100.0%)
16	Was there a statement disclosing the source of funding for the study?	3	0

#### The quality of HTA projects based on the Drummond Checklist

Again, only those with the economic section were assessed by this checklist [20]. The first seven questions of the Drummond checklist are about the study design, which other than determining the economic significance of the study question, most of the reports had no answers for these questions. Regarding the method of data collection (questions 8–21), all the reports had reported the outcomes and costs. However, the relevant details had been scarcely presented. The remaining questions deal with the analysis and interpretation of results. These two followed a similar pattern in which the details had been barely stated, and only the final result had been presented (Table 11).

**Table 11:** Assessment results based on the Drummond Checklist

No.	Drummond’s questions	The number of reports that had taken this step
1	The research question is stated	9 (69.2%)
2	The economic importance of the research question is stated	5 (38.5%)
3	The viewpoint(s) of the analysis are clearly stated and justified	7 (53.8%)
4	The rationale for choosing alternative programmes or interventions compared is stated	8 (61.5%)
5	The alternatives being compared are clearly described	8 (61.5%)
6	The form of economic evaluation used is stated	13 (100.0%)
7	The choice of form of economic evaluation is justified in relation to the questions addressed	10 (76.9%)
8	The source(s) of effectiveness estimates used are stated	13 (100.0%)
9	Details of the design and results of effectiveness study are given (if based on a single study)	2 (15.4%)
10	Details of the methods of synthesis or meta-analysis of estimates are given (if based on a synthesis of a number of effectiveness studies)	4 (30.8%)
11	The primary outcome measure(s) for the economic evaluation are clearly stated	13 (100.0%)
12	Methods to value benefits are stated	0
13	Details of the subjects from whom valuations were obtained were given	0
14	Productivity changes (if included) are reported separately	0
15	The relevance of productivity changes to the study question is discussed	0
16	Quantities of resource use are reported separately from their unit costs	13 (100.0%)
17	Methods for the estimation of quantities and unit costs are described	11 (84.6%)
18	Currency and price data are recorded	12 (92.3%)
19	Details of currency of price adjustments for inflation or currency conversion are given	6 (46.5%)
20	Details of any model used are given	12 (92.3%)
21	The choice of model used and the key parameters on which it is based are justified	7 (53.8%)
22	Time horizon of costs and benefits is stated	4 (30.8%)
23	The discount rate(s) is stated	4 (30.8%)
24	The choice of discount rate(s) is justified	2 (15.4%)
25	An explanation is given if costs and benefits are not discounted	3 (23.08%)
26	Details of statistical tests and confidence intervals are given for stochastic data	2 (15.7%)
27	The approach to sensitivity analysis is given	6 (46.1%)
28	The choice of variables for sensitivity analysis is justified	5 (38.5%)
29	The ranges over which the variables are varied are justified	3 (23.1%)
30	Relevant alternatives are compared	9 (69.2%)
31	Incremental analysis is reported	9 (69.2%)
32	Major outcomes are presented in a disaggregated as well as aggregated form	6 (46.1%)
33	The answer to the study question is given	13 (100.0%)
34	Conclusions follow from the data reported	13 (100.0%)
35	Conclusions are accompanied by the appropriate caveats	10 (76.9%)

#### The quality of HTA projects based on the Preferred Reporting Items for Systematic review and Meta-Analysis (PRISMA) Checklist [21]

Based on Table 12, most reports had used the systematic review method. However, none had used the words ‘systematic review’ or ‘meta-analysis’ in its report title. Only 16 (69.57%) reports had prepared an executive summary from their report. A small number of studies (34.78%) had specified their structured question. The methods section also showed that none of them had addressed the existence of an executive protocol at the beginning of their study and 4.35% of the reports had examined the risks of possible error. In the results section, (69.57%) of reports had explained the method of selecting articles. However, only 13.04% had presented the results of outcomes synthesis. The discussion sections of most studies contained weaknesses and a few of the reports had covered the items highlighted by PRISMA.

**Table 12:** Assessment results based on the Preferred Reporting Items for Systematic review and Meta-Analysis (PRISMA) Checklist

PRISMA questions			The number of reports that had taken this step
Title		1. Identify the report as a systematic review, meta-analysis or both	0
Abstract		2. Provide a structured summary	16 (69.6%)
Introduction	Rationale	3. Describe the rationale for the review in the context of what is already known	12 (52.2%)
	Objectives	4. Provide an explicit statement of questions being addressed with reference to participants, interventions, comparators, and outcomes (PICO)	8 (34.8%)
Methods	Protocol and registration	5. Indicate if a review protocol exists, and if and where it can be accessed	0
	Eligibility criteria	6. Specify study characteristics (e.g. PICO, length of follow‐up) and report characteristics (e.g. years considered, language, publication status) used as criteria for eligibility, giving rationale	9 (36.1%)
	Information sources	7. Describe all information sources (e.g. databases with dates of coverage, contact with study authors to identify additional studies) in the search and date last searched	17 (73.9%)
	Search	8. Present full electronic search strategy for at least one database, including any limits used, such that it could be repeated	14 (60.9%)
	Study selection	9. State the process for selecting studies (i.e. screening, eligibility, included in systematic review and, if applicable, included in the meta‐analysis)	16 (69.6%)
	Data collection process	10. Describe method of data extraction from reports (e.g. piloted forms, independently, in duplicate) and any processes for obtaining and confirming data from investigators	11 (47.9%)
	Data items	11. List and define all variables for which data were sought (e.g. PICO, funding sources) and any assumptions and simplifications made	10 (43.5%)
	Risk of bias in individual studies	12. Describe methods used for assessing risk of bias of individual studies (including specification of whether this was done at the study or outcome level), and how this information is to be used in any data synthesis	9 (36.1%)
	Summary measures	13. State the principal summary measures (e.g. risk ratio, difference in means)	3 (13.0%)
	Synthesis of results	14. Describe the methods of handling data and combining results of studies, if done, including measures of consistency (e.g. I2) for each meta‐analysis	3 (13.0%)
	Risk of bias across studies	15. Specify any assessment of risk of bias that may affect the cumulative evidence (e.g. publication bias, selective reporting within studies)	1 (4.3%)
	Additional analyses	16. Describe methods of additional analyses (e.g. sensitivity or subgroup analyses, meta-regression), if done, indicating which were pre‐specified	5 (21.7%)
Results	Study selection	17. Give numbers of studies screened, assessed for eligibility, and included in the review, with reasons for exclusions at each stage, ideally with a flow diagram	16 (69.6%)
	Study characteristics	18. For each study, present characteristics for which data were extracted (e.g. study size, PICO, follow-up period) and provide the citations	13 (56.5%)
	Risk of bias within studies	19. Present data on risk of bias of each study and, if available, any outcome level assessment	7 (30.4%)
	Results of individual studies	20. For all outcomes considered (benefits or harms), present, for each study, (1) simple summary data for each intervention group, (2) effect estimates and confidence intervals, ideally with a forest plot	4 (17.4%)
	Synthesis of results	21. Present results of each meta-analysis done, including confidence intervals and measures of consistency	3 (13.0%)
	Risk of bias across studies	22. Present results of any assessment of risk of bias across studies	3 (13.0%)
	Additional analysis	23. Give results of additional analyses, if done (e.g. sensitivity or subgroup analyses, meta-regression)	3 (13.0%)
Discussion	Summary of evidence	24. Summarise the main findings including the strength of evidence for each main outcome; consider their relevance to key groups	7 (30.4%)
	Limitations	25. Discuss limitations at study and outcome level (e.g. risk of bias) and at review-level (e.g. incomplete retrieval of identified research, reporting bias)	4 (17.4%)
	Conclusions	26. Provide a general interpretation of the results in the context of other evidence, and implications for future research	5 (21.7%)
Funding	Funding	27. Describe sources of funding for the systematic review and other support (e.g. supply of data); role of funders for the systematic review	0

## Discussion

The current study was conducted to identify the methods of dissemination of HTA reports and their effects from the perspective of researchers and stakeholders. Among 23 HTA reports that were conducted between the years 2008 and 2013, four were related to pharmaceutical technologies and 19 were related to medical equipment technologies. Medical equipment technologies are much more expensive and are more likely to be requested for technology assessment by the Article 20 Commission and it is clear that the HTA results have influenced the decisions of the Article 20 Commission.

Based on our findings, the rate of utilisation of HTA results among various stakeholders was varied and on the whole was not desirable. What follows is a discussion of the results based on the domains of the Payback model, with a final discussion on the barriers of implementation of HTAs.

### The knowledge advancement impact of HTA reports

In this study, we examined the knowledge advancement impacts of HTA reports from the researchers’ perspective and appraised the quality of HTAs to assess the knowledge advancement impact of HTA reports. From these steps we found that the most common way to disseminate the results of the HTA projects was publication and that all reports had been published in the form of a book by MOHME, with their full texts also being available in the HTA Office website. However, owing to inappropriate notification of the HTA Office, a minimal number of researchers and stockholders were aware of this. Other reasons for low awareness of HTA reports were lack of familiarity with MOHME and interaction with stakeholders, lack of dissemination of results in the user’s language and minimal publication of HTA articles in well-known journals. Of the questionnaires sent to the PIs of the 23 conducted HTAs, 20 researchers stated that they had published 15 articles from 12 HTAs, 11 articles were indexed in PubMed or Scopus, and the average citation was 2.42. The fact that half of the HTAs have not been published in articles is a case to ponder; this may be due to many different reasons, most worryingly, the low quality of the assessments conducted, which was specifically addressed by the stakeholders in the interview.

The scarcity of evidence, the low budget allocated to research execution, inappropriate professional ties between those prioritising HTA topics with executive or end-user units from researcher’s perspective, and barriers in identification and prioritisation of topics from the target audience’s perspective were also other reasons for the scarce publishing of HTA reports.

Examining the quality of HTAs also showed that most of the reports were focused on determining the target population of the technology, the description of the technology, the necessity of using the technology in comparison to similar existing technologies, and the cost effect of using the technology.

However, none of the reports presented a clear introduction of the research team, the harm and hazard of using the technology for specialists, public and the environment, or the direct effect of the technology on quality of life. Regarding the methods sections of HTAs, a small number of studies (34.78%) had specified their structured question, few of the reports had examined the possible errors, and only 13.04% had presented the results of outcomes synthesis. The discussion sections of most studies also contained weaknesses. Therefore, insufficient presentation of relevant details in HTA reports is another reason for the low rate of publishing of HTA reports.

The lack of publishing of HTA reports as a result of the low quality of the HTAs can itself affect the utilisation of their results. Earlier studies have shown that the quality of health technology assessments and research in Iran is an important barrier toward its implementation [22]; however, this is an issue in other countries as well.

In our study, appraisal by the Drummond and QHES checklists showed that the relevant details had been scarcely presented about outcomes, costs and analysis, with report scores (QHES) ranging from 38 to 87, with a mean of 72 (SD 15.25) from 100. To our knowledge, no study has investigated the quality of HTA reports; however, those that have assessed economic evaluations have shown that, in most Australian reports (before 1978), the reason for selecting the control group was unknown and most articles had not performed marginal analysis. Therefore, in the absence of marginal analysis, the fitness of cost-effectiveness ratio comparisons of the two groups was questionable [23]. On the other hand, Iran’s economic evaluation studies (between 1999 and 2012) do not cover most of the country’s health problems and are of low quality and suffer from major methodological problems; therefore, these studies cannot be a source of information for decision-makers [24]. Thailand’s economic evaluation studies (between 1982 and 2005) also showed the dire need to focus on the quality of reports and their analyses. Additionally, the absence of economic evaluation publications for 15 out of 20 cases of Thailand’s major problems indicate the inappropriate distribution of research resources for determining cost-effective interventions to reduce the burden of disease [25]. Most published Korean studies had not observed international standards, their designs were faulty, their types and perspectives had not been clearly described in most cases, the duration was short, and only half of the studies had performed sensitivity analysis [26].

Since the benefit of economic reports is directly related to the quality of studies, and economic evaluation studies only prove beneficial to policy-makers if they are correctly conducted and accurately reported, studies recommend the further development of national guidelines for conducting and reporting economic evaluation studies, determining the comprehensive and systematic method of selecting and prioritising evaluation topics [25], conducting economic evaluations on regional priorities, and training researchers and policy-makers in the estimation and utilisation of economic evaluation data [24].

### The capacity-building impact of HTA reports

Evaluation of capacity-building from the researchers’ perspective revealed a wide range of results. The respondent researchers had declared that the assessment had led to the acquisition of new skills (such as conducting systematic reviews and/or economic evaluations) and facilitated the securing of research grants from other organisations. However, the status of capacity-building in the other cases was not desirable. Only one assessment had been considered an applied research and had garnered scores from MOHME, which is in itself contrary to the nature of HTAs (according to the current rules, projects that lead to changes in decision-making are revealed in a central committee in MOHME and, if the committee approves the impact of the project, it is assigned a score; this score is considered a plus point for the promotion of the investigator and the organisation in which the investigator is working in). As a project that has the ability to directly affect decision-making, an HTA should garner special points for the researchers conducting it. In the assessed HTAs, it is not clear whether the researchers have attempted to obtain scores or have not succeeded in attaining them; yet, it is clear that the evaluation of knowledge-generating organisations (universities and research centres) and evaluation of academic members should consider a special score for documentations that can be directly used in decision-making and the score should not be dependent on the adoption of a decision in the health system. There are two main reasons behind this; firstly, scientific evidence is considered a factor affecting policy-making at the macro levels of the health system (which is why this is referred to as ‘evidence-informed’ policy-making, and not ‘evidence-based’). Therefore, we cannot expect the decisions adopted to be entirely based on scientific evidence, including those of HTAs. Secondly, HTAs are utilised in different policy-making organisations, e.g. the Article 20 Commission, the Medical Equipment Office, the Food and Drug Organisation, and insurance companies. Currently, tracing the impact of assessment results regularly and continuously has not been possible, even in MOHME and the HTA Office. Thus, how can one expect the researcher to foresee and register the implementation of the HTAs they have performed? Under the current circumstances two solutions can be taken into consideration. HTA projects should be assigned the same score as applied researches are (both for individual assessment and organisational assessment), and – as the steward of HTA in the country – the HTA Office and/or National Institute of Health Research should follow the implementation of the assessment results and hand over their documentations to the researchers.

Stakeholder engagement in the development of HTA reports can also improve the following of impact of assessment results. The respondent researchers had declared that technology providers and policy-makers providing and allocating finances for technology had the highest rate of participation in preparing the HTA reports. However, the highest rate of stakeholder engagement was at the time of extracting the conclusions. Perhaps active stakeholder engagement from the early phases of assessment would be more useful.

Nine out of 20 assessments (45%) had facilitated the securing of research grants from other organisations (inside and/or outside the Health System). The reason this question was mentioned in the capacity-building section is that, when a high quality HTA is conducted for an organisation, others begin to trust the quality of the researcher’s work, hence facilitating future orders. The interpretation of these numbers should be done with caution, because, overall, the number of HTAs conducted is limited. However, the number of researchers conducting these assessments is also limited. Therefore, on the whole, it seems that this part of capacity-building needs further research. Furthermore, the researchers of nine assessments declared that their project results were used to define future research projects. Bearing in mind the nature of HTAs, this finding is not far from expectation, as the assessments conducted by an individual are different. However, this matter can be viewed through a different perspective. Every time an assessment is conducted, new research questions are put forth [2]; its root cause can be traced to the knowledge gap observed, the need for new data to estimate costs, and the need to examine the other aspects of technology (apart from effectiveness and cost-effectiveness, which are currently being done). However, the fact that none of these needs become the topic of the next research should be addressed. The most important cause, which itself can have many causes, is that the researchers conducting the HTA are not active in a specific line of research; this issue warrants further study.

### The impact of HTA reports on decision-making

The impact of HTA report results at the various levels of policy-making was assessed according to the researcher and audience’s perspective.

The researchers believed that the greatest impact had been on policy-makers providing and allocating finances (seven of the 20 HTA projects’ findings had resulted in a change in the policy-makers providing and allocating finances), despite the fact that this group of stakeholders are the first-line users of assessment results in Iran. Why have all the 20 projects not caused a change in the aforementioned policy-makers? The reason may be that, basically, the researcher is unaware of the utilisation of such assessment results in the first place (as mentioned in the capacity-building session), or that the assessment result has not been delivered to the policy-maker and/or even if they are aware, they have not utilised it in decision-making. Bearing in mind the interview results, it seems that policy-makers’ lack of awareness of assessment results is the most important cause that requires special consideration. Moreover, as stated in the previous sections, the most common method of dissemination was the publication of articles and delivery of complete project reports or executive summaries to potential end-users, which has been performed in only half the assessments. Therefore, it could be said that the HTA results do not reach the stakeholders, so one cannot expect them to be utilised.

The respondent researchers mentioned that companies importing or producing technology and policy-makers regulating technology-related research and innovation needs had been least affected by the HTA reports; however, it seems that the question about the rate of creating change in these stakeholders based on HTA results was not appropriate and the data was not valid, as these groups are the target audiences that researchers have the least access to.

The impacts of HTA reports from the perspective of the HTA reports also showed that some organisations claimed that they would have used the results if they had had access to their results; yet, some stakeholders believed they could not utilise the results in any case. Barriers in stewardship, identification and prioritisation of topics, performance and dissemination of HTA results were the main reasons for the low implementation of HTAs from the target audience’s perspective. With regards to the dissemination of HTA results, some researchers meant ‘executive summaries’ not ‘policy briefs’. Therefore, researchers not only do not provide results in the language of policy-makers, but also they are not appropriately familiar with the methods of transferring the results correctly.

The rate of utilisation of HTA results and its determinant factors have been investigated in a small number of studies. Two separate studies in England examined the effects of the HTA programme using the Payback framework through the years 1993–2003 and 2003–2013 [2, 27]. The results of these studies are not entirely parallel to ours, because in Iran’s HTA programme there are only secondary and cost-effectiveness studies, while England’s HTA programme is a combination of primary and secondary studies. In Iran, 0.75 articles had been published from each assessment, whereas this figure was 3.36 in the two English studies. Overall, 57% of the secondary English studies had affected policy-makers in the HTA programme and 10% had led to behaviour change. In Iran, however, under the best possible conditions, 35% of the assessments have led to change in policy-makers providing and allocating technology finances and 15% have affected technology providers. The most important determinant factors of the assessment results in England were the timeliness of results, the quality of research, communications with stakeholders and the existence of specific clients for assessment results. The most important reason behind the lack of impact of assessment results was not being ready on time.

Another study that has examined the impact of HTA programmes in the Netherlands observed the following impacts from 34 assessments: 101 peer-reviewed articles, over 25 doctoral theses, referral to the results of six assessments in guidelines and the implementation of novel medical strategies in 11 assessments [28].

### The barriers of utilisation of HTA results

Barriers of implementation of HTAs were extracted from the researchers and target audience’s perspectives. From their point of view, one of the most important barriers of utilisation of assessment results is that the service-providing organisation (MOHME) is independent of the organisation providing finances (the Ministry of Welfare and Social Security). In addition to the other effects it has through different methods, this division has led to a negative attitude toward the independence of the HTA programme (cost and content wise), and a lack of consistency of the two organisations’ priorities for conducting assessments [29]. Other barriers mentioned by researchers and target audiences were:

- The research needs of insurance organisations (which are subdivisions of the Ministry of Welfare and Social Security) are not taken into consideration when preparing the HTAs.
- The average time of performing an HTA was 1 year, which is long considering the time limitations in decision-making. The lengthiness of the duration of HTA is a barrier that has been addressed in earlier studies [22] and mentioned here as well. However, one stakeholder believed that the reasons for the lengthy process is not the time for conducting the assessment itself, but the time elapsed from announcing the topic to signing the contract and the duration of peer review and approval of the final report. The lengthy nature of the bureaucracy procedure has also been underscored in other studies [2].
- All the assessments had been funded by the HTA Office, although this was not agreed upon by all the stakeholders. Insurance organisations believed that stakeholders should pay for the costs of the assessments; this budget should be saved in a common fund and be used for conducting HTAs. From their point of view, on the one hand, this will make the assessments impartial, and on the other, the considerations relevant to different stakeholders will be taken into account when conducting the assessments.
- Noteworthy is that, among the 20 reports for which the researchers had completed the questionnaires, 19 researchers had reported that political factors had no effect on the delivery of results. This indicates the impartiality of the researchers and is very desirable, although upon interviewing the stakeholders we found this impartiality to be confounded in certain cases.
- Some (40%) PIs believed that the budget provided for HTA was not proportionate to the volume of work. Nevertheless, researchers’ opinions may differ in on this issue; however, the important point is that, if the budget is insufficient, the quality of the assessments will be greatly affected by, for example, reduced stakeholder engagement (e.g. the presence of clinical specialists and technology experts in the HTAs is necessary, but may be prevented by the scarcity of budget). Moreover, systematic reviews that need to be conducted by two independent reviewers and/or the appropriate collection of technology costs will be affected by the low budget and will not be performed properly.
- Here, we found that the researchers could not specify the technology stakeholders comprehensively; the ‘policy-makers regulating educational and research needs relevant to technology’ and ‘patients and the public’ had not been introduced as stakeholders in almost half of the assessments. This indicates that, although it is necessary to teach the researchers how to identify their research stakeholders, organisations in charge of HTA should themselves take responsibility for identifying the stakeholders and this responsibility should not be handed over to the researchers. Bearing in mind the scarcity of the types of technologies, and subsequently the consistency of their stakeholders, it seems that this would be a doable task. This identification should take place at the beginning of the assessment and the researchers should be informed to raise the possibility of active participation of appropriate stakeholders in the assessment. Furthermore, by identifying these stakeholders, the active dissemination at the end of the study will be facilitated [22].

Although the active engagement of all the stakeholders in the assessment seems impossible, depending on the type of technology, the participation of specific stakeholders is vital; something which has been neglected by researchers.

Garrido et al. [30] believe that the effects of HTA could be more valuable when key stakeholders (patients, providers, industry, health management, health professionals, payers and government) become involved in this process as much as possible and decision-makers become committed to using HTA reports (and technology assessment meets their needs), the resources required to implement existing decisions are provided, the process of evaluation and decision-making is transparent, and knowledge and skills are passed on to relevant stakeholders.

A systematic review of barriers of the use of evidence by policy-makers reported a range of factors like poor contact, collaboration and relationships, a lack of access to research, poor dissemination, a lack of relevant research, the poor quality of research, having no time or opportunity to use research evidence, policy-makers and other users not being skilled in research methods, and policy-makers’ beliefs about the utility of evidence use, as the most frequently reported barriers of evidence use. Poor long-term policy planning and inflexible and non-transparent policy processes in developing countries were also other barriers [31].

Three important points arose regarding the evaluation of the HTA programme’s impact in Iran. Firstly, considering that this study evaluates only the first 23 HTA reports produced, it is highly likely that the situation has improved in recent years. Second, the most important limitation in accurately recording the impact of HTA reports is the poor documentation of the client organisations (or individuals) and meeting documents in stakeholder organisations. Therefore, the only method for capturing the impact is to conduct interviews and use individuals’ memories. Finally, the most difficult part of capturing the impact of the HTA report is when the result of decision was one that was supposed to be decided (regardless of the result of the HTA report). For example, without regard to the result of the HTA report, technology should be allowed to enter, now if the result of the report is positive, because there is no discussion about it, it will not remain in memory and the risk of information bias would increase.

## Conclusions

With regards to knowledge advancement impact of HTA reports, the current study’s findings showed that publication of HTA projects was the most common step taken to disseminate the results. With regards to impact on capacity-building, conducting the HTA led to an improvement in researchers and their colleagues’ new skills and facilitated the securing of research grants from other organisations. From the quality aspect, most reports used the systematic review method but the relevant details had scarcely been presented related to outcomes, costs and analysis. The greatest impact observed of HTA reports on decision-making were on policy-makers’ provision and allocation of finances; companies importing or producing technology and policy-makers regulating technology-related research and innovation needs had been the least affected by the HTA reports and none of the HTA results had been utilised in policy-making outside the health system. Barriers in stewardship, identification and prioritisation of topics, performance and dissemination of HTA results were the main barriers of implementation of HTAs.

To advance the goals of the HTA programme in Iran, the barriers identified in this and other studies should be thoroughly considered and solutions should be actively applied.

## Additional file

Additional file 1: Researcher’s questionnaire. (DOCX 29 kb)

## Abbreviations

HIC: Higher Insurance Council
HTA: health technology assessment
INAHTA: International Network of Agencies for Health Technology Assessment
MOHME: Ministry of Health and Medical Education
PRISMA: Preferred Reporting Items for Systematic review and Meta-Analysis
QHES: The Quality of Health Economic Studies Instrument
