# Correction to: Impact assessment of Iran’s health technology assessment programme

**DOI:** 10.1186/s12961-018-0369-y

**Published:** 2018-09-07

**Authors:** Bahareh Yazdizadeh, Farideh Mohtasham, Ashraf Velayati

**Affiliations:** 1 Knowledge Utilization Research Centre (KURC), Tehran University of Medical Sciences, Tehran, Iran; 2 Shahid Beheshti University of Medical Sciences, Tehran, Iran

## Correction to: Yazdizadeh et al. Health Research Policy and Systems (2018) 16:15 DOI 10.1186/s12961-018-0286-0

It has been highlighted that the original article [1] contains a typesetting mistake in affiliation 2.

This was incorrectly captured as Shaheed Beheshti University of Medical Sciences. This correction article shows the correct and incorrect affiliation:

Incorrect affiliation:

- Shaheed Beheshti University of Medical Sciences, Tehran, Iran

Correct affiliation:

- Shahid Beheshti University of Medical Sciences, Tehran, Iran
