# Supplementary material for: Impact assessment of Iran’s health technology assessment programme
Source: Health Res Policy Syst. 2018 Feb 22;16:15. doi: 10.1186/s12961-018-0286-0 (PMC5824575; doi:10.1186/s12961-018-0286-0)
Supplement: Supplementary file 1 — Researcher’s questionnaire. (DOCX 29 kb) [file 12961_2018_286_MOESM1_ESM.docx]

**Additional File 1: Researcher’s Questionnaire**

**The ‘Assessing the Impact of HTA Reports in Iran’ Questionnaire**

| Dear Mr. / Ms. ……….,  We hereby inform you that a project titled “Assessing the Impact of Health Technology Assessment Reports in Iran” is underway in Tehran University of Medical Sciences’ *Knowledge Utilization Center.*  In this study, the impacts of HTA reports prepared between September 2008 and September 2013 will be investigated through a case study approach. Furthermore, the questionnaires completed by the HTA s’ principle investigators will be examined using desk analysis methods.  Therefore, we kindly request you -as the principle investigator of the project- “………………….” to complete the questionnaire. This questionnaire examines the impact of HTA reports in four domains of knowledge dissemination activities, knowledge advancement, capacity-building and impact on decision-making.  Please let us know your valuable comments if any clarification is needed in the questionnaire. For further information, please call Ms. … at (021)88975658.  Thank you |
| --- |

| Title of Health Technology Assessment (HTA): ………………………..  Principle investigator of HTA: ……………………….. |
| --- |

1. **HTA Specifics**

Please complete the specifics of the HTA in this section:

- 1. HTA co-investigators and their affiliations:

| **Principle investigator and co-investigators** | **Affiliation** |
| --- | --- |
|  |  |
|  |  |
|  |  |
|  |  |
|  |  |
|  |  |
|  |  |
|  |  |

- 1. Date of signing the HTA contract
  2. Date HTA finished
  3. Has active engagement of different stakeholders been employed in preparing the HTA report? (first specify the stakeholders, then specify their type and level of engagement)

| **Stakeholders** | **They are stakeholders (yes/no)** | **They have participated (yes/no)** | **Level of participation** | | | |
| --- | --- | --- | --- | --- | --- | --- |
|  |  |  | **Proposal development** | **Execution** | **Analysis** | **Conclusion** |
| Technology providers (including Physicians, pharmacists, nurses, the National Medical council, the Food and Drug Organization etc.) |  |  |  |  |  |  |
| Companies importing or producing technology |  |  |  |  |  |  |
| Policy-makers providing and allocating finances to technology, including, the insurance office, the Medical Equipment office, supervision and funding of curative affairs office, the Iranian Parliament’s health commission, Ministry of Health and Medical Education’s (MOHME) policy-making council, policy-makers and the hospital management and clinical governance office of MOHME, and policymakers at MOHME’s standardization and tariff office |  |  |  |  |  |  |
| Policy-makers regulating technology-related research and innovation needs, including deputies of research and technology, universities of medical sciences etc. |  |  |  |  |  |  |
| Policy-makers regulating technology-related educational needs including deputies of education |  |  |  |  |  |  |
| Public, patient associations etc. |  |  |  |  |  |  |
| Other stakeholders: ………………………………….. |  |  |  |  |  |  |

- 1. Has the current HTA been financially supported by the HTA Office? If your reply is ‘no’ please explain who the sponsor was. Yes □ No □
  2. How much was the budget of this project?
  3. Were the financial resources provided for the report sufficient?

Yes □ No □

- 1. Have political factors and outside pressure have affected the report result? Yes □ No □

1. **Dissemination**
   1. What measures have you and your colleagues taken to implement the results of the aforementioned project (by you and/or the HTA Office)? (you may choose more than one option)

| □ | 1. The publication of articles in domestic scientific-research journals |
| --- | --- |
| □ | 1. Publication of articles in international scientific-research journals |
| □ | 1. Presentation in domestic conferences, workshops and seminars |
| □ | 1. Presentation in international conferences, workshops and seminars |
| □ | 1. Delivery of the complete or summarized reports to their potential end-users |
| □ | 1. Displaying the complete or summarized reports in the website to allow access to their potential end-users |
| □ | 1. Dissemination of HTA results in non-scientific publications (such as magazines or newspapers read by the public) |
| □ | 1. Dissemination of results in newsletters and bulletins (such as intra-organizational publications that publish the scientific and non-scientific news related to that organization) |
| □ | 1. Presenting the assessment results to media journalists (radio, television) and/or giving interviews |
| □ | 1. Setting up meetings with potential stakeholders to introduce the assessment results |
| □ | 1. Development and delivery of results in a language appropriate to the target audiences (such as simple writings for patients and/or the public, short reports for managers and authorities) |
| □ | 1. Other steps that lead to the delivery of assessment results to their target audiences |
| □ | 1. None of the aforementioned |

1. **Knowledge advancement**
   1. Please enter the titles of the articles that you have published from the aforementioned project in the table below.

| **Row** | **Article title** | **Journal name** | **Year of publication** |
| --- | --- | --- | --- |
|  |  |  |  |
|  |  |  |  |
|  |  |  |  |

1. **Capacity-building**

4-1 Has the project been considered an applied one and garnered points from MOHME? Yes □ No □

(an HTA is considered a client-based project and may garner points for the research centers or individuals if implemented)

4-2 Have you or the co-investigators acquired new skills (such as systematic review and or economic assessment) in order to design future studies? Yes □ No □

4-3 Has this project facilitated the securing of research grants from other organizations (inside or outside the health system)? Yes □ No □

4-4 Have the results of this project been utilized by you or other researchers to define future research projects? (This does not include citations)

Yes □ Number: ………………… No □ I don’t know □

4-5 Was the project or part of the project an academic thesis?

Yes □: 1. Masters’ thesis □ 2. M.D. thesis □ 3.MPH (Master of Public Health) thesis □ 4.Post-doctoral thesis □

No □

4-6 How many students have graduated through the aforementioned project? ………… students

4-7 Has part of the project costs been used to strengthen your organization’s research resources (such as software and equipment)? Yes □ No □

4-8 If your answer to the previous question is ‘yes’, what percentage of the budget has been spent on strengthening resources?

Less than 10% □ 10-25% □ 26-50% □ More than 50% □

4-9 Have part or all of the infrastructures required for this project been provided by mechanisms other than the projects own costs? Yes □ No □

1. **Impact on decision-making**

5-1 HTA results can be utilized at any level of policy-making of health services (e.g. national, regional, administrative, managerial or professional). Have the results of your assessment been utilized at any level of policy-making and brought about any changes?

| **Stakeholders** | **Created change** | | |
| --- | --- | --- | --- |
|  | Yes | No | I do not know |
| Technology providers (including Physicians, pharmacists, nurses the National Medical Council, the Food and Drug Organization etc.) | □ | □ | □ |
|  |  |  |  |
| Companies importing or producing technology | □ | □ | □ |
|  |  |  |  |
| Policy-makers providing and allocating finances to technology, including, the insurance office the Medical Equipment office, supervision and funding of curative affairs office, the Iranian Parliament’s health commission, MOHME is policy-making council, policy-makers and the hospital management and clinical governance office of MOHME, and policymakers at MOHME’s standardization and tariff office | □ | □ | □ |
|  |  |  |  |
| Policy-makers regulating technology-related research and innovation needs, including deputies of research and technology, universities of medical sciences etc. | □ | □ | □ |
|  |  |  |  |
| Policy-makers regulating technology-related educational needs including Deputies of Education | □ | □ | □ |
|  |  |  |  |
| Public, patient associations etc. | □ | □ | □ |
|  |  |  |  |
| Other stakeholders: ………………………………….. | □ | □ | □ |
|  |  |  |  |

5-2 Do you expect your HTA results to be utilized in future policy-makings and decision-makings? Yes □ No □

5-3 Have the results of the aforementioned project been utilized in any of the following studies or documentations?

| **Row** | **Type of study** | **Please name the documentation if your reply is ‘yes’** |
| --- | --- | --- |
|  | Systematic reviews  Yes □ No □ I don’t know □ |  |
|  | Service delivery guidelines (Clinical guidelines or public health guidelines)  Yes □ No □ I don’t know □ |  |
|  | Educational content for patients and/or the public (Patient decision aids, etc.)  Yes □ No □ I don’t know □ |  |
|  | Policy brief (Policy brief is a documentation that is prepared to help make decisions about the possible options of policy-making and includes scientific evidence on the advantages and disadvantages of various policy-making options)  Yes □ No □ I don’t know □ |  |
|  | Policy documents, guidelines and/or executive organizations’ legislations  Yes □ No □ I don’t know □ |  |

5-4 Have the results of the project been utilized in compiling a book?

Yes □ No / I don’t know □

Please name the book and give its year of publication if your answer is ‘yes’.

…………………………………………………………………………………………………………………………………………………………………………………………………………………………………………………………………………………………………………

5-5 Have the results of the aforementioned project been used in the development of educational content for professional groups (continuing education and/or education of academic students)

Yes □ No / I don’t know □

Please name the educational content if your answer is ‘yes’:

…………………………………………………………………………………………………………………………………………………………………………………………………………………………………………………………

5-6 Have the results of the aforementioned project been used in policy-makings outside the health system directly or indirectly)?

Yes □ No / I don’t know □ Not applicable □

Please describe how they have been used if your answer is ‘yes’:

…………………………………………………………………………………………………………………………………………………………………………………………………………………………………………………………

5-7 Have the project results been utilized in your university and/or local policy-making?

Yes □ No / I don’t know □ Not applicable □

Please describe how they have been used if your answer is ‘yes’:

…………………………………………………………………………………………………………………………………………………………………………………………………………………………………………………………

1. **Comments**

6-1 If your HTA has not been utilized as expected, what do you think the reasons are for the lack of its utilization?

…………………………………………………………………………………………………………………………………………………………………………………………………………………………………………………………………………………………………………………………………………………………………………………………………………………………………………………………………………………………………………………………………………

6-2 If you have any further comment to give on your HTA, please write it down.

……………………………………………………………………………………………………………………………………………………………………………………………………………………………………………………………………………………………………………………………………………………………………………………………………………………………………………………………………………………………………………………

Thank you for taking time to complete the questionnaire.
